# Minutes of the Second Annual Meeting of the Ohio State Dental Society

**Published:** 1868-02

**Authors:** G. W. Keely


### Proceedings of Societies.

### MINUTES OF THE SECOND ANNUAL MEETING OF THE OHIO STATE DENTAL SOCIETY.

COLUMBUS, Ohio, Dec. 24th, 1867.

Society met pursuant to adjournment, at the "Rooms of the Young Mens' Christian Association," at 2 o'clock P. M. (A quorum not being present, no regular morning session was held.)

President Dr. Geo. Watt, called the society to order. The Secretary being absent, G. W. Keely was appointed *pro tem.* Minutes of the last semi-annual meeting were read and approved. Roll of members being called, the following answered to their names: G. W. Keely, Geo. Watt, J. Taft, R. Corson, A. A. Blount, G. W. Dunn, B. T. Spellman, N. W. Williams, J. Fowler, J. W. Wortman, D. R. Jennings, C. H. Harroun, J. B. Beauman.

Drs. Harroun and Jennings were appointed to fill the vacancy in the Committee on membership.

The following was offered, "At the next meeting I will propose that the By-laws be so amended as to provide that the Annual Meeting shall be held on the first Tuesday of December in each year, and that the semi-annual meetings be discontinued.

Signed, A. A. BLOUNT."

A letter from Dr. A. Berry was read, regretting his inability to be present, which was received, and ordered placed on file.

Drs. Blount, Dunn and Corson, were appointed to propose candidates for officers to be elected for the ensuing year. Report received and laid on the table. Drs. Williams and

Corson were appointed to audit the Treasurer's account, and reported the same correct, with a balance of $50 12 in the Treasury.

Committee on Membership recommended Drs. J. H. Paine, of Middletown, and G. L. Paine, of Xenia, for active members; and Dr. S. C. Taylor, of Toledo, as an honorary member, all of whom were duly elected.

Report of nominating committee taken up, and the following officers were elected:

*President*—J. Taft.

1*st Vice-President*—C. H. Harroun.

2*d Vice-President*—C. R. Butler.

*Corresponding Secretary*—N. W. Williams.

*Recording Secretary*—A. W. Maxwell.

*Treasurer*—A. Berry.

Dr. J. Taft, President elect, took the chair, making a few appropriate remarks. On motion of Dr. Watt, the members elected at the last semi-annual meeting be required to pay but one-half the yearly dues.

On motion, the By-laws fixing the time for the annual meeting was suspended by unanimous vote, after which it was—*Resolved,* To hold the next annual meeting commencing at 10 o'clock A. M., on the first Tuesday of December.

The following resolution was submitted by Dr. Spellman, and passed.

That any member now in debt to this society, who shall continue so until the next meeting, shall appear in person or by proxy at the same, and show cause for delinquency, and those failing to do so shall cease to be members; and that the Treasurer be requested to notify delinquents.

The rubber question was taken up, and discussed fully by Dr. J. Taft, and spoken upon by Keely, Spellman, Harroun, and others; and a determination shown to contest the matter to the end.

Society then adjourned to meet at 7 o'clock P. M.

G. W. KEELY, *Sec. pro tem.*

Evening session—Society met, President Taft in the chair. Minutes of the afternoon session read and approved. On motion, Drs. Watt and J. H. Paine were appointed to select delegates to the American Dental Association. The following were proposed, and duly appointed: Drs. B. A. Rose, J. W. Wortman, John Fowler, H. M. Edson, W. R. Lilley, A. Terry, H. H. Harrison, A. W. Maxwell, H. C. Howells, R. Corson, T. W. French, L. Buffett.

The Bill to "regulate the Practice of Dentistry in the State" was taken up, and discussed by Drs. Taft, Spellman, Keely, Paine, Watt, Harroun, and others. When on motion, Drs. Harroun, Watt and Jennings were appointed to select a committee of fifteen, whose duty it shall be to look after the interest of this bill at the coming session of the General Assembly. The following were chosen: J. Taft, G. W. Keely, F. H. Whitslar, W. P. Horton, M. De Camp, A. A. Blount, J. B. Beauman, A. W. Maxwell, F. H. Rehwinkle, W. M. Harriott, J. H. Paine, C. H. Harroun, G. L. Paine, H. R. Smith, J. M. Rhodes.

The Executive Committee reported the following subjects for discussion:

*First*—Necrosis of Alveolar Process, cause and treatment.

*Second*—Preparation of Cavities for filling.

*Third*—Means for controlling flow of saliva.

*Fourth*—Dental Medicines.

*Fifth*—Mechanical Dentistry.

The first subject was discussed in a very *spirited* manner by Drs. Watt and Spellman, and spoken upon with much interest by others.

The second subject was then taken up and discussed at considerable length by Drs. Harroun, Keely, Taft, Blount, and a few others.

It being late, and the design of the society to adjourn this evening, the other subjects were laid over.

The President announced the Standing Committees for the ensuing year as follows:

*Executive Committee*—M. De Camp, J. W. Wortman, J. Fowler.

*Membership*—B. T. Spellman, C. H. Harroun, J. H. Paine.

*Dental Ethics*—N. W. Williams, F. H. Rehwinkle, G. W. Keely.

*Publication*—Geo. Watt, G. L. Paine, A. A. Blount.

On motion, the President appointed the following Essayists for the next meeting: Drs. H. A. Smith, A. Berry, C. R. Butler, J. H. Paine, C. H. Harroun.

The following was adopted: *Resolved*, That the Automatic Plugger submitted for examination by Dr. S. C. Taylor of Toledo, is in our opinion superior to any one heretofore exhibited to this society.

J. Taft's bill of $22.50 for printing, and a bill of $5.00 for the use of Hall, was allowed and ordered paid.

Although but few members were present, all felt their time had been profitably spent, and "that it was good to be here." After a short season of social intercourse the society adjourned.

G. W. KEELY, *Sec'y, pro tem.*

